# Supplementary material for: Favorable Preclinical Pharmacological Profile of a Novel Antimalarial Pyrrolizidinylmethyl Derivative of 4-amino-7-chloroquinoline with Potent In Vitro and In Vivo Activities
Source: Biomolecules. 2023 May 14;13(5):836. doi: 10.3390/biom13050836 (PMC10216263; doi:10.3390/biom13050836)
Supplement: Supplementary file 1 [file biomolecules-13-00836-s001.zip › biomolecules-2303677-supplementary.pdf]

Basilico N. et al . Favorable preclinical pharmacological profile of a novel antimalarial pyrrolizidinylmethyl derivative of 4-amino-7-chloroquinoline with potent in vitro and in vivo activities

Supplementary information

Chemistry

Table S1. Optimization of the final step of **MG3** synthesis: study of the best conditions of reaction

Amine (eq)	Quinoline (eq)	Phenol (eq)	K_2CO_3 (eq)	Solvent	T(°C)	T (h)	Y (%)
3	1			none	120	6	73
2	1			none	120	5	81
1	1	1		none	120	4	65
1	1	2		none	120	4	59
2	1		1	isopropanol	80	24	9
2	1		2	n-Butanol	120	24	47
1	1		2	Toluene	110	24	15
1	1		1	N-Methyl-2-pyrrolidone	120	6	35

Amine= (hexahydro-1*H*-pyrrolizin-7a-yl)methanamine (**5**)
Quinoline= 4,7-dichloroquinoline
Y= yield

Analytical RP-HPLC

Hitachi Elite LaChrome system consisting of the following components:
Pump: Hitachi L-2130
Autosampler: Hitachi L-2200
Column Oven: Hitachi L-2300
UV/VIS diode array detector: Hitachi L-2450:
Software: EZChrom Elite 3.1.7
Stationary phase Purospher® STAR RP-18 endcapped (5 µm) LiChroCART® 250-4
Eluent A: Phosphate Buffer pH 6
Eluent B: MeOH
Elution mode: Gradient (see Table below)
Column temperature 40 °C
Flow rate: 1 mL/min
Wavelength: 254 nm
Gradient:

t (min)	%B
0-5	60
5-8	70
8-10	70
10-12	80
12-15	80

Figure S1. HPLC analysis of **MG3**

Preparation Method A

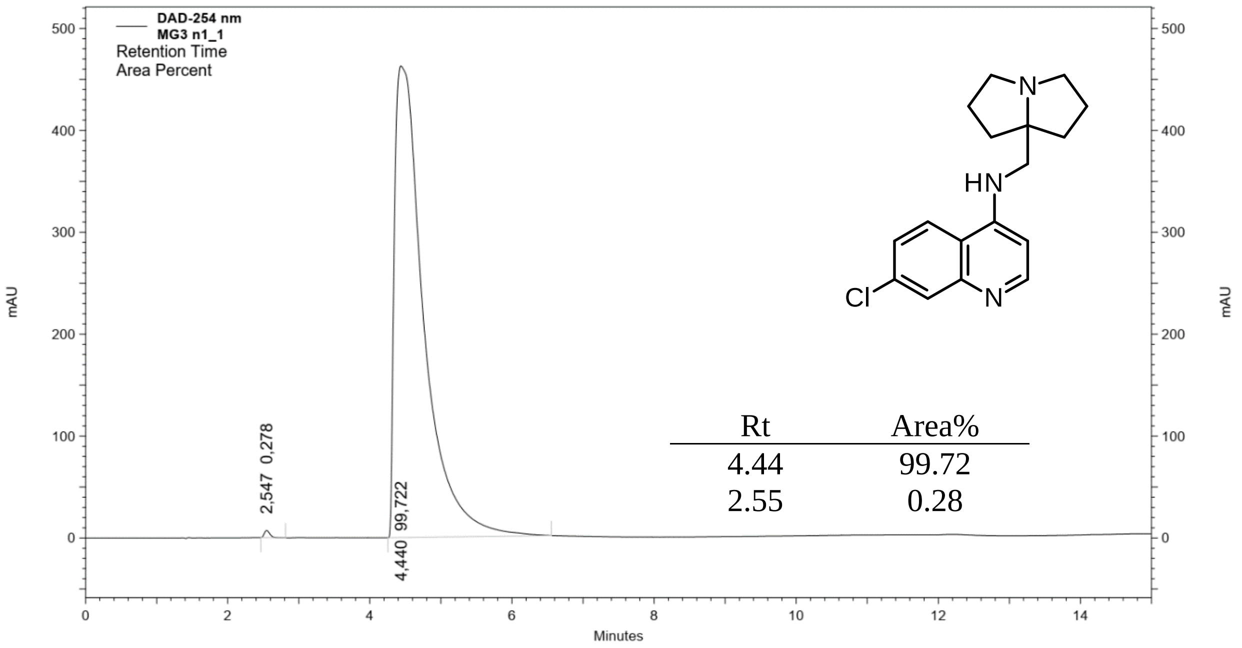

Preparation Method B

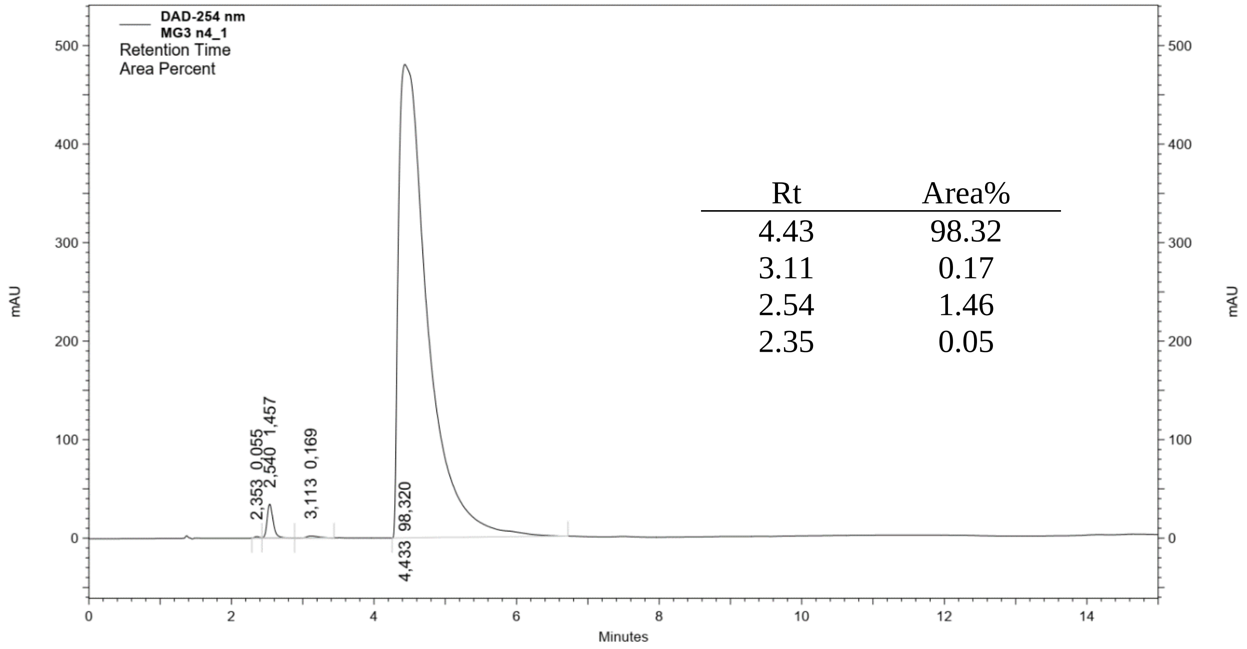

Figure S2. ^{1}H NMR analysis of **MG3**

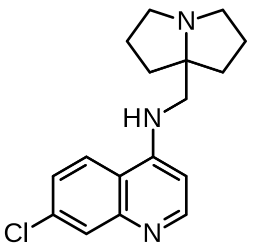

Preparation Method A

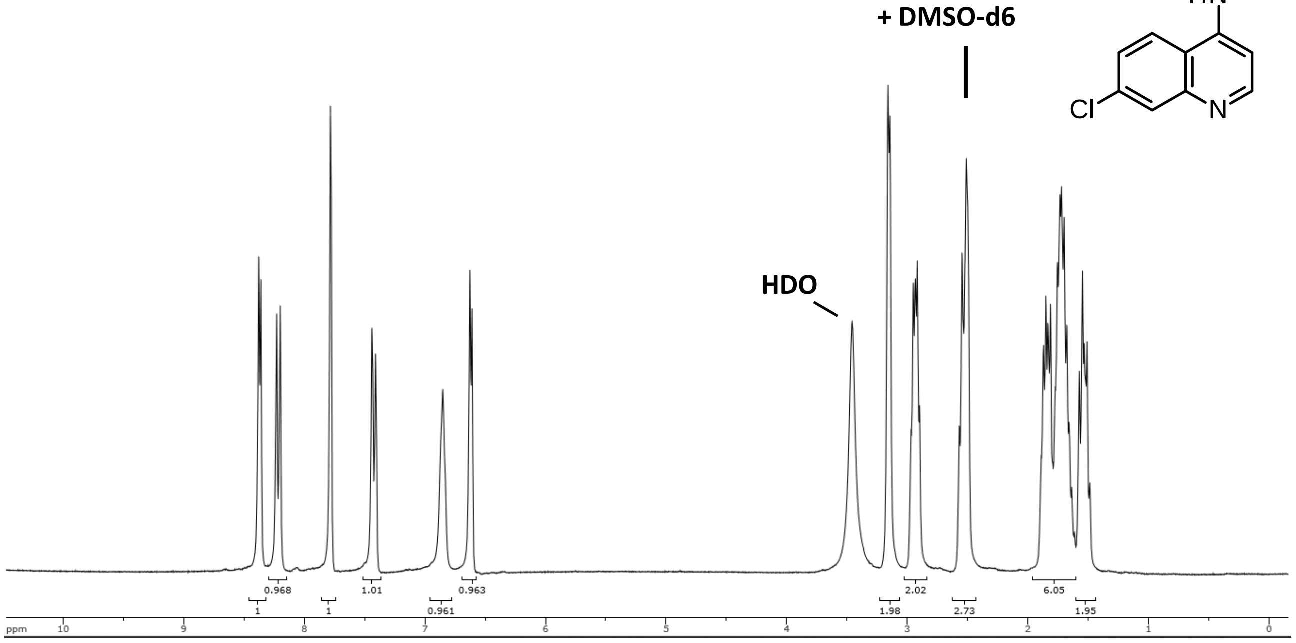

Preparation Method B

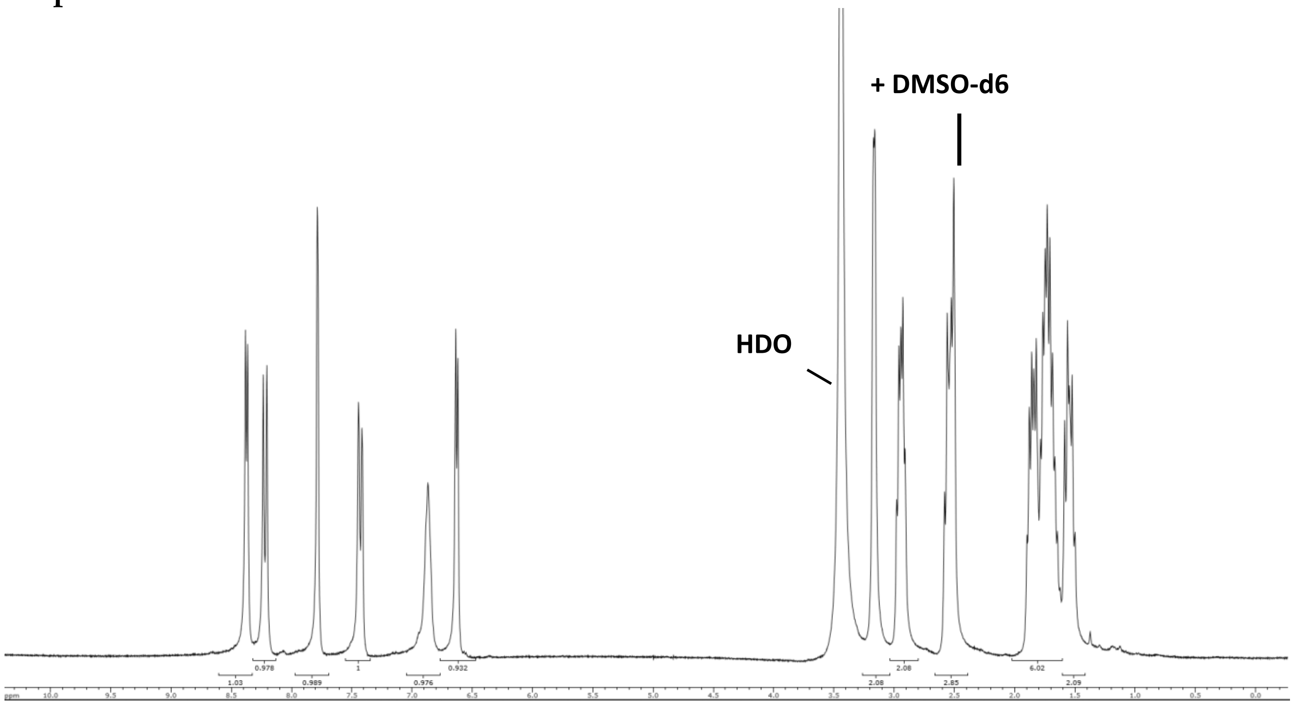

Figure S3. COSY NMR analysis of **MG3**

Preparation Method A

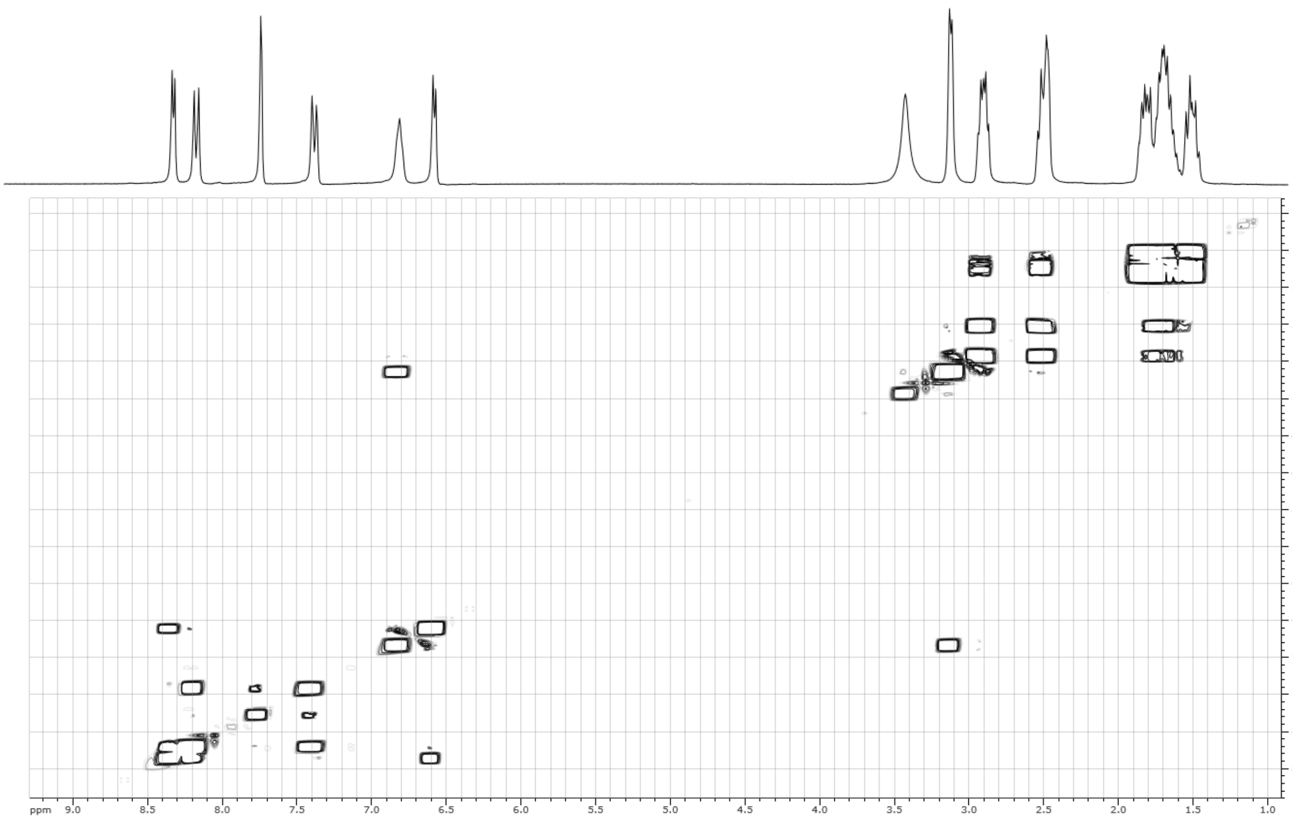

Preparation Method B

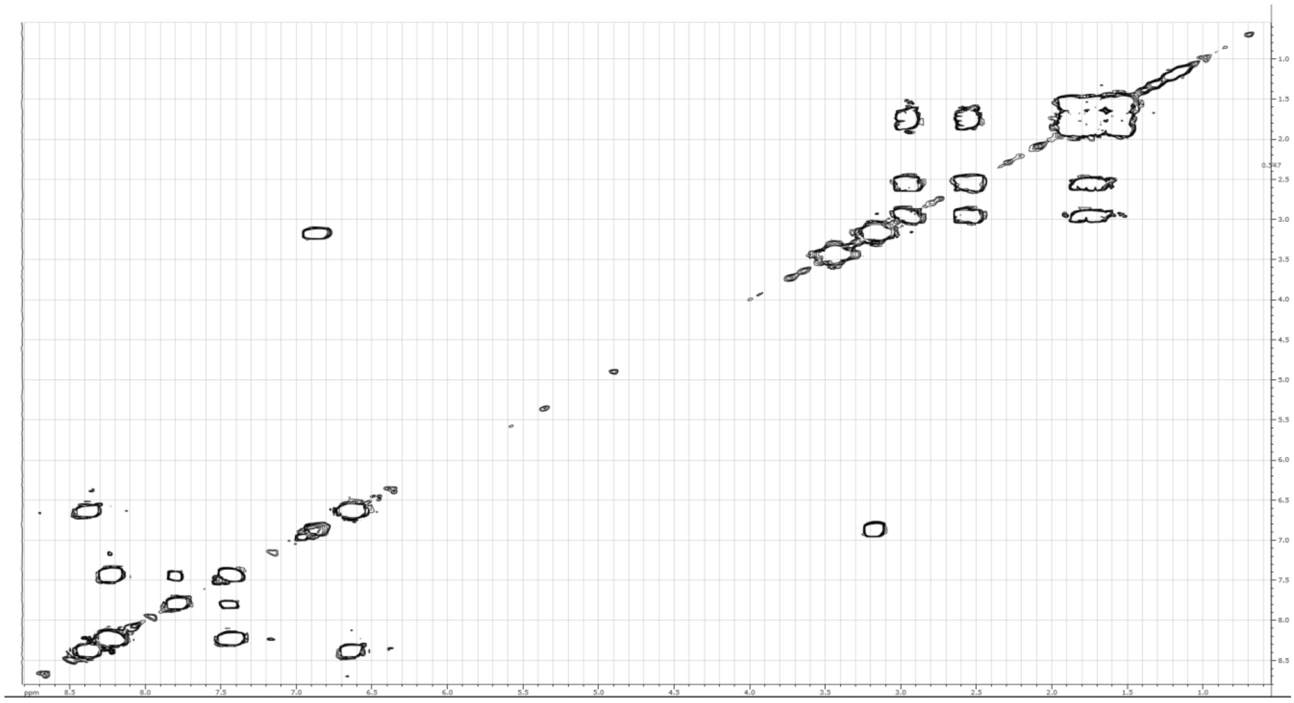

Figure S4. ^{13}C NMR analysis of **MG3**

Preparation Method A

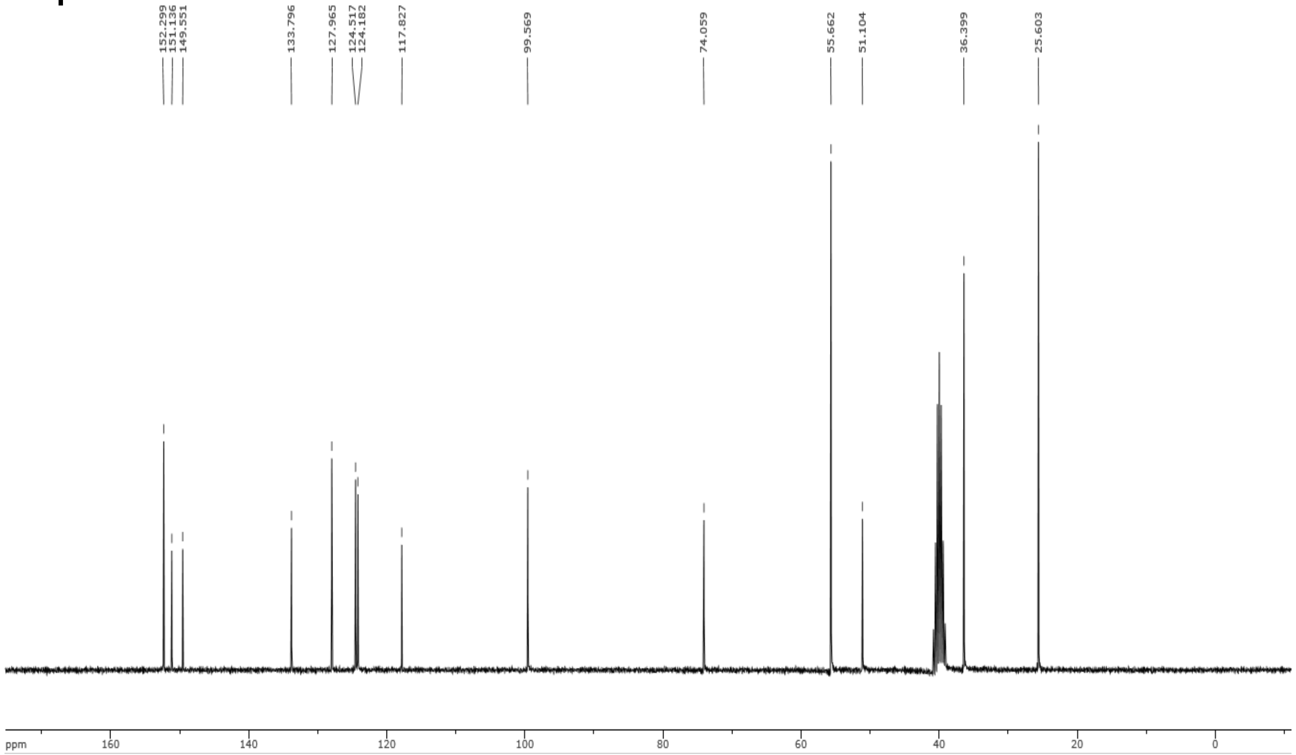

Preparation Method B

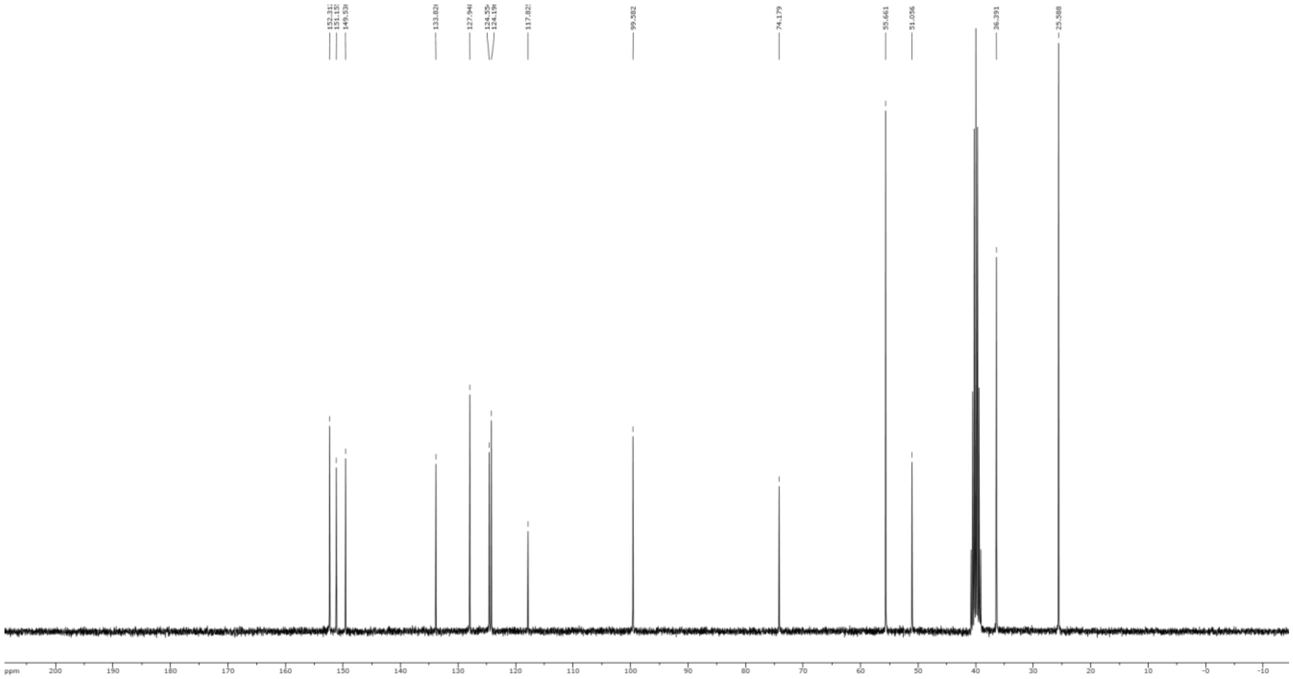

In vitro combination study

To ascertain if **MG3** could be used in combination therapy, experiments were conducted in vitro testing **MG3** in combination with azithromycin, DHA, artesunate or the tetraoxane RK182 [O'Neill 2010] using the potentiation tests previously described [Benoit-Vical F et al, 2000]. CQ was used as control. Isobolograms were constructed by plotting a pair of fractional IC_{50} values for each combination of **MG3** or CQ with the companion drugs. An isobologram close to the diagonal indicates an additive effect. Curves significantly (>2) above or below the diagonal indicate antagonistic or synergistic effects, respectively [Vivas, L et al 2007; Bell A et al 2005].

As shown in **Fig S5**, an additive interaction was observed between **MG3** and azitromycin against both the K1 (CQ-R) and the 3D7 (CQ-S) strains. Moreover, the combination **MG3** with artesunate, DHA or the tetraoxane RKA-182 as companion drugs resulted also additive with a non-significant trend toward antagonism against both CQ-R(K1) and CQ-S (3D7) strains (**Table S2** and **Fig. S6**).

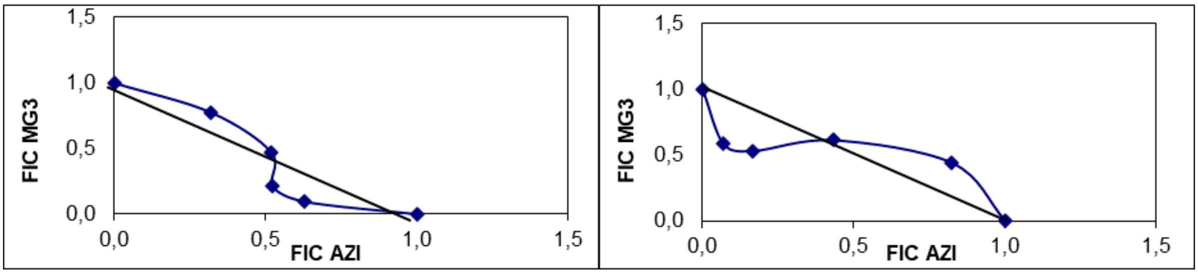

K1 3D7

Figure S5. Isobologram analysis of the antimalarial activity of **MG3** in combination with azitromycin against K1 (CQ-R) and 3D7 (CQ-S) strains

Table S2. Mean ∑FIC of the interactions between **MG3** and RKA-182, artesunate or DHA *in vitro* against *P. falciparum* 3D7 and K1 at the IC_{50} level.

	**Mean ∑FIC₅₀ (95% CI)**	
**Drug**	**3D7**	**K1**
	**MG3**	
**RKA-182**	1.45 (1.17 – 1.73)	1.24 (0.85 – 1.63)
**ASN**	1.43 (1.30 – 1.55)	1.25 (0.94 – 1.56)
**DHA**	1.49 (1.33 – 1.65)	1.25 (1.21 – 1.29)
**Drug**	**CQ**	

**RKA-182**	-	1.19 (0.77 – 1.60)
**ASN**	-	1.67 (1.44 – 1.90)
**DHA**	-	1.27 (1.16 – 1.38)

ASN: artesunate; DHA: dihydroartemisinin; CQ: chloroquine

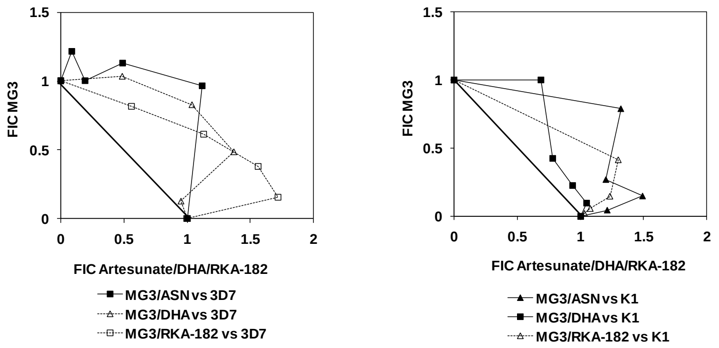

Figure S6. Isobologram analysis of the antimalarial activity of **MG3** in combination with DHA or RKA182 against 3D7 (CQ-S) (left panel) or K1 (CQ-R) (right panel) strains. An isobologram close to the diagonal indicates an additive effect. Curves significantly (>2) above or below the diagonal indicate antagonistic or synergistic effects, respectively.

***In vitro* activity of MG3 against *P falciparum* sexual gametocyte stages**

The in vitro activity of **MG3**, CQ and methylene blue on *Pf* gametocytes form was evaluated in the luminescent transgenic 3D7 strain (3D7elo1-pfs16-CBG99) at two different stages (I-III and V) using the methodology previously described (D'Alessandro S. et al. 2016). Briefly, 100 μL of culture medium were removed from each well to increase hematocrit; 70 μL of resuspended culture were transferred to a black 96-well plate; 70 μL of D-luciferin (1 mM in citrate buffer 0.1 M, pH 5.5) were added. Luminescence measurements were performed after 10 min with 500 ms integration time using a Synergy4-BioTek reader. CQ and Methylene Blue (MB) were used as control. As expected for this class of compounds, the IC_{50} of **MG3** against young, stage I-III gametocytes was around 100 nM, like CQ, but the activity decreased against mature stage V gametocytes (**Table S3**). Methylene Blue maintained its potency versus both stages (D'Alessandro S et al 2013)

Table S3: *In vitro* transmission blocking activity of **MG3** on *P. falciparum* gametocytes

	Pf-3D7 gametocytes[a] IC_{50} (nM)	
	>95% Stage I-III	>95% Stage V
MG3	101.6 ± 22.1	10681.3 ± 2794.7
CQ	104.1 ± 34.7	11739.7 ± 4118.7
Methylene Blue	59.6 ± 21.7	144.6 ± 42.6

[a]Pf gametocytes from the transgenic 3D7elo1-pfs16-CBG99 strain were used at both stage I-III and stage V. Data are expressed as Mean ± S.D. of three independent experiments performed in duplicate. Methylene Blue is used as positive control.

In vivo activity of MG3

MG3 was not curative when given at 30 mg/kg for 3x to *P. berghei* infected mice, but it was better than CQ, amodiaquine (AQ) or artesunate in prolonging the mice survival (22 vs. 13 vs.11 vs 8 days, respectively). On day 4 **MG3** and AQ inhibited parasitaemia by 100%, not significantly different than CQ or ASN (**Table S4**)

Table S4. Oral curative activity of **MG3** against *P. berghei* ANKA.

Compound/drug	Oral dose mg/kg	Average survival (days) after infection	% Suppression of parasitaemia (on day 4 post infection)
**MG3**	3 x 30	22 (17, 23, 23, 24, 24)	100
Chloroquine	3 x 30	13 (12, 12, 13, 13, 13)	99.84
Amodiaquine	3 x 10	11 (9, 11, 11, 12, 13)	100
Artesunate	3 x 30	8 (7, 8, 8, 9, 9)	99.09
Untreated Control (no drug)	-	6 (6, 6, 6, 6, 6)	-

Figure S7. Metabolite profile of MG3 after 1 hour incubation at the concentration of 10 μM with mouse, rat, dog, monkey, and human hepatocytes, versus control.

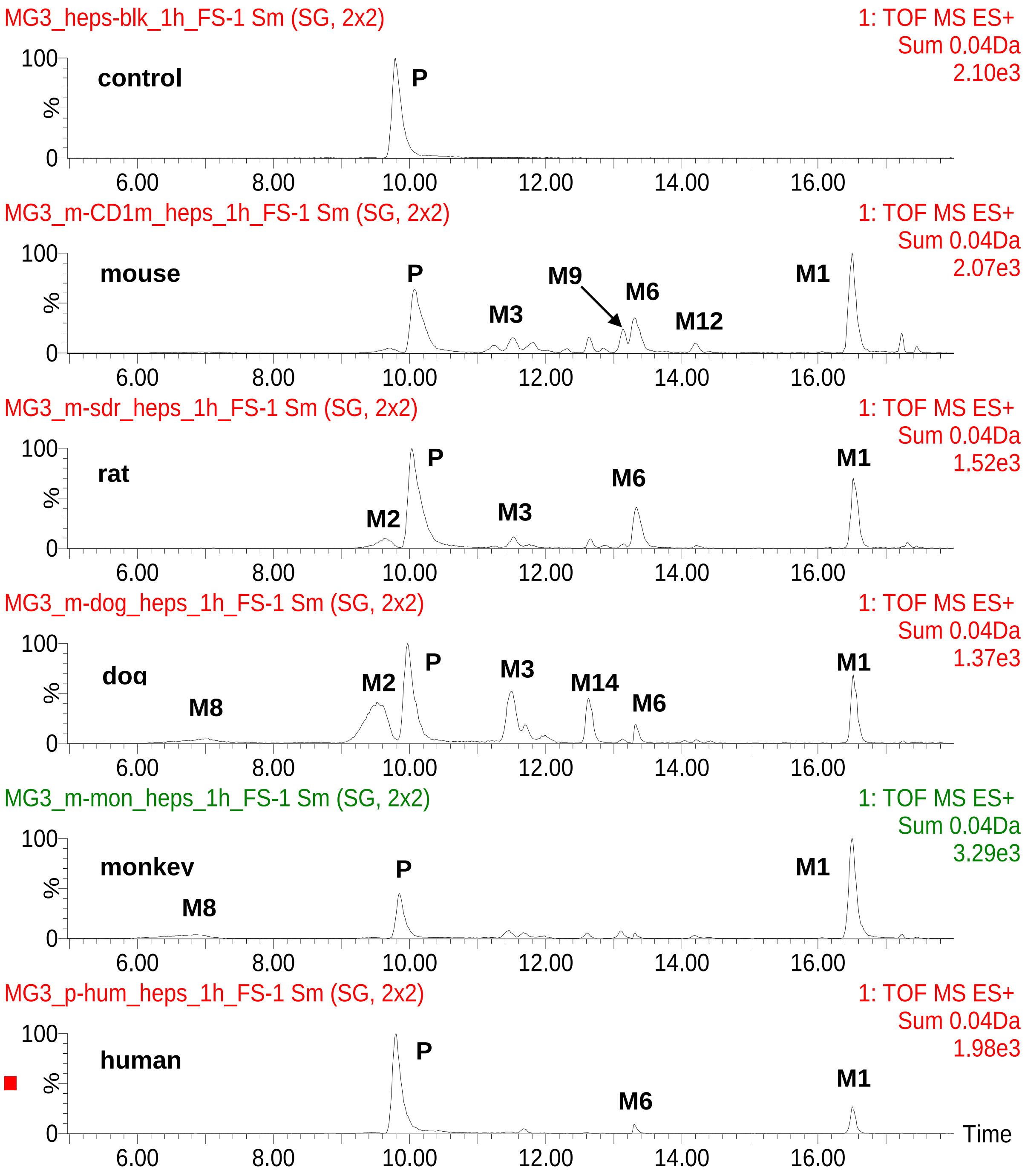

Figure S8. MS/MS spectra and structures of MG3 and metabolite M1

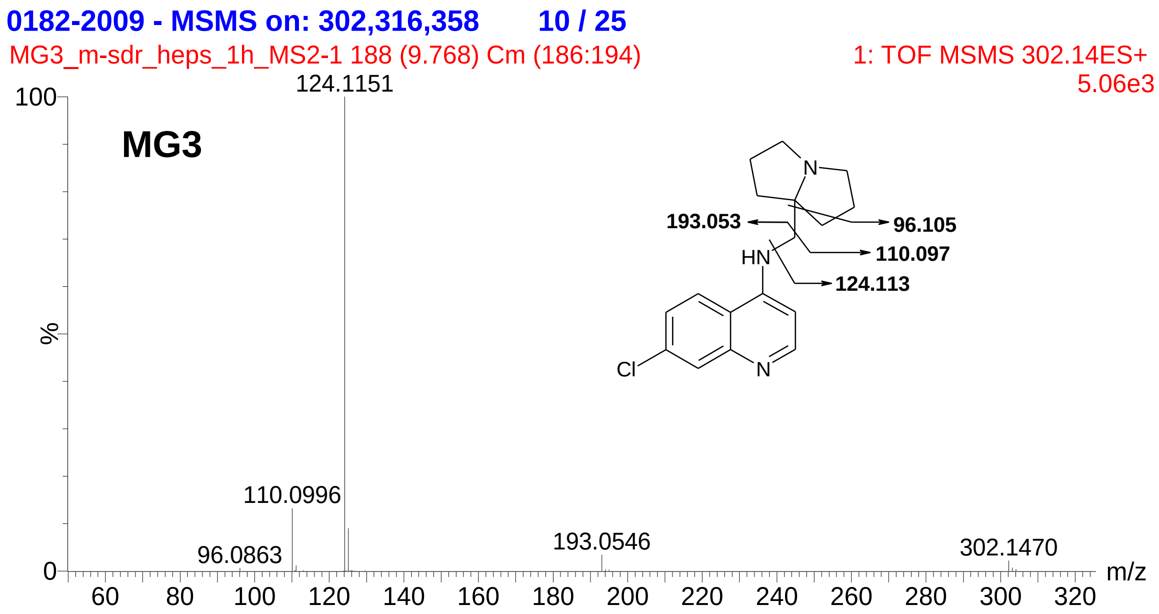

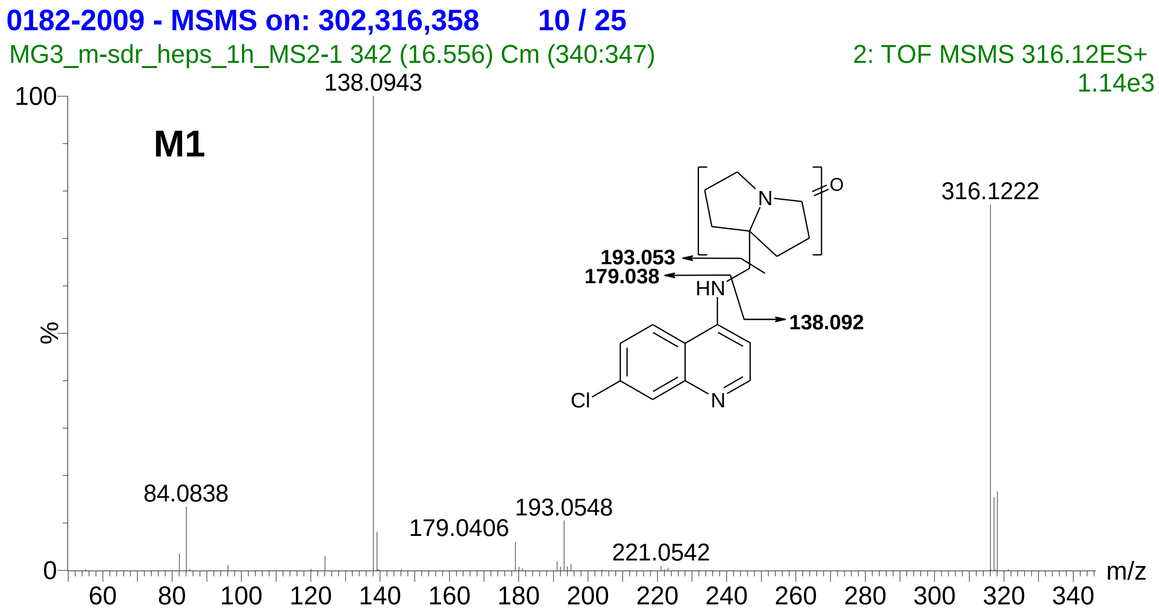

Figure S9. MS/MS spectra and structures of metabolites M2 and M3.

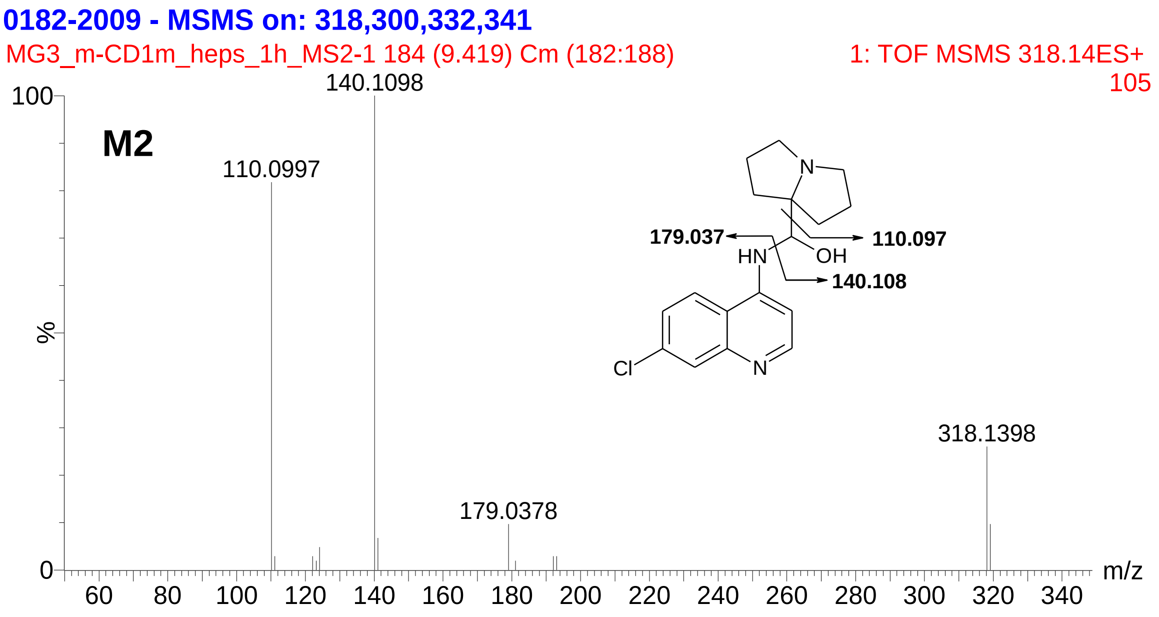

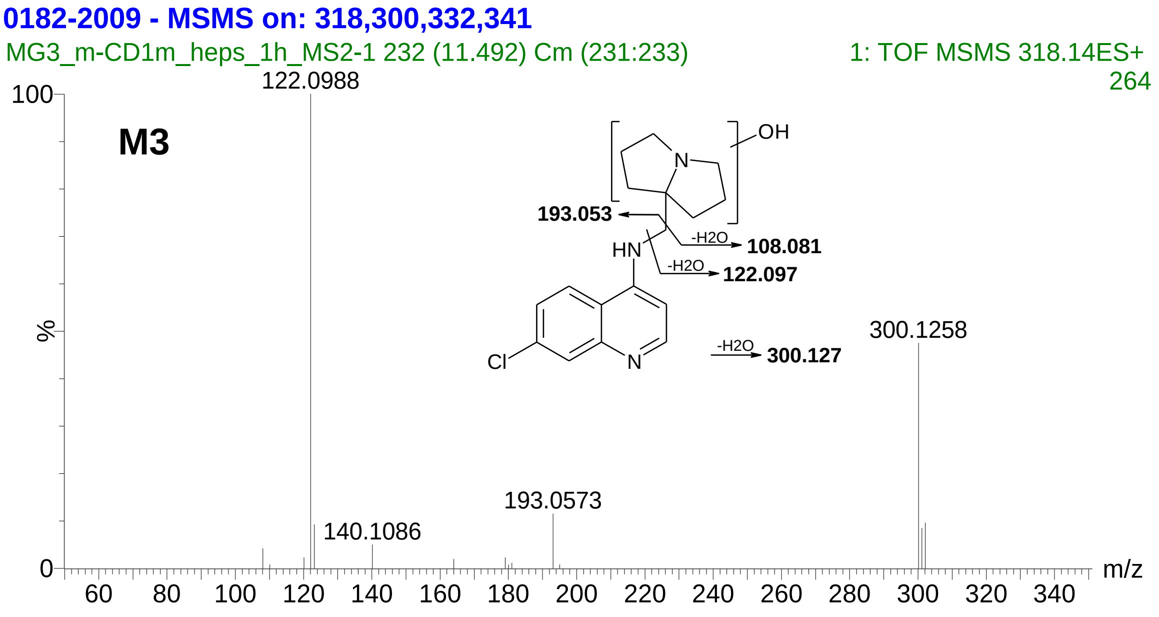

Figure S10. MS/MS spectra and structures of metabolites M6 and M8.

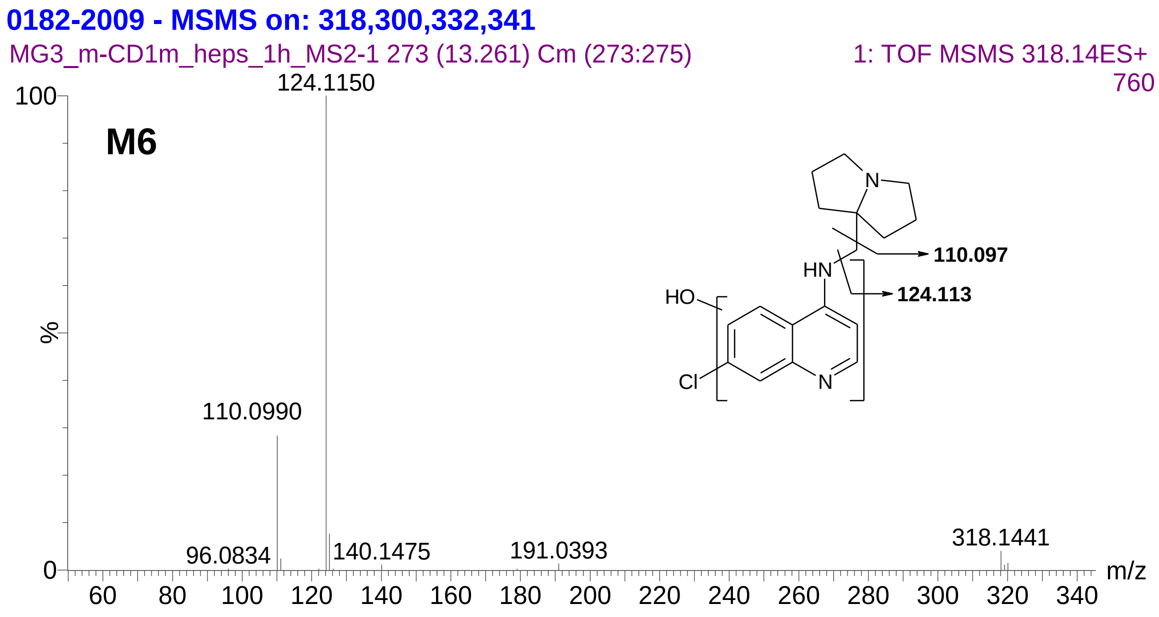

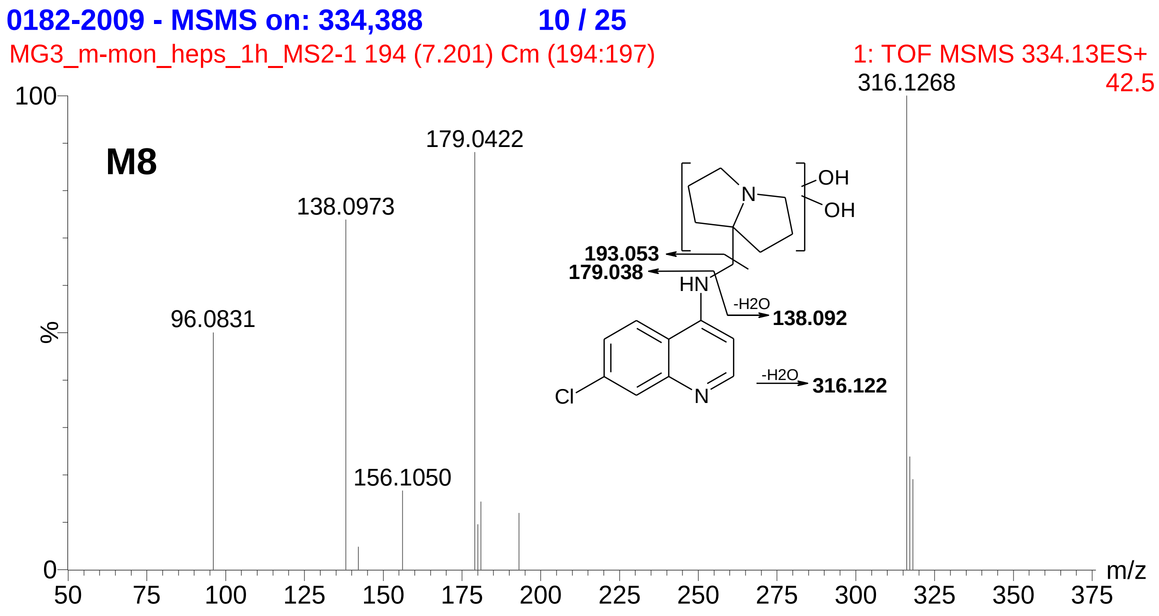

Bacterial Reverse Mutation Assay

The compound **MG3** was evaluated for its potential to induce reversion mutations in the *Salmonella typhimurium* tester strains TA98, TA100 and in *Escherichia coli* strain WP2 uvrA. Mutation test was carried out using the preincubation method (30 min, 37°C) with and without phenobarbital-5,6-benzoflavone induced rat liver metabolic (S9) activation (Mortelmans, K et al 2000). **MG3** was dissolved in distilled water (H_2O) at the maximum concentration of 40 mg/mL and tested in a preliminary range-finding test in all strains of *Salmonella typhimurium* and WP2 uvrA at doses ranging from 1.95 to 1000 µg/plate with and without metabolic activation to determine the toxicity of the compound. Based on the results of the range–finding test, **MG3** was tested starting from 15.63 µg/plate to 1000 µg/plate with the preincubation method, with and without S9 fraction. The results of the study of **MG3**, with or without exogenous rat liver S9 used to induce metabolic activation, demonstrated no significant, dose-related, and reproducible increases in revertant colony numbers at any dose tested in all the bacterial strains (**Table S5**). Therefore, **MG3** was judged to be non-mutagenic.

Table S5: MG3 Bacterial Reverse Mutation Assay (with metabolic activation): summary of fold increase value (ratio treated revertant numbers/ solvent revertant numbers)

	TA 100		TA 98		WP2 uvrA	
Dose µg/plate	Range finding	I° Exp.	Range finding	I° Exp.	Range finding	I° Exp.
1000	0.7 T	0.8 T	1.0	1.1	0.8	0.7 S
500	1.2	0.8 K	1.2	1.5	0.7	0.8 K
250	1.0	0.8	1.2	1.4	0.7	1.5
125	1.0	0.6	1.3	1.2	0.9	1.4
62.5	1.0	0.6	1.3	1.1	0.7	1.4
31.25	1.0	ND	1.0	ND	0.8	ND
15.63	0.9	ND	1.2	ND	0.7	ND
7.81	1.2	ND	1.5	ND	0.9	ND
3.9	1.1	ND	0.9	ND	0.9	ND
1.95	1.1	ND	0.9	ND	1.0	ND
Positive Control	7.7	5.1	26.5	37.2	8.2	13.0

Positive controls:
2-aminoanthracene 1.25 µg/plate for TA100 and TA98
2-aminoanthracene 5 µg/plate for WP2 uvrA

I° Exp.: I° Experiment; T: toxicity; K: Marginally Toxicity ; ND : Not determined

Methods for hERG interaction

Membranes were prepared from HEK293 cells stably transfected with the human ERG (ether-a-go-go related gene; U04270) from Cytomix. [3H]-astemizole (PerkinElmer Life Sciences, Specific Activity: 86 μCi/nmol) was used as radioligand. Non-specific binding was evaluated in the presence of an excess of 10 μM cold astemizole. The concentration-response curve was performed in a range from 0.03 to 30 μM (seven concentrations). Binding experiments were made at 22°C for 60 min in triplicate [Chiu PS et al. 2004].

References for Supplementary

1. O'Neill, P., Amewu, R., Nixon, G., Bousejra ElGarah, F., Mungthin, M., Chadwick, J., Shone, A., Vivas, L., Lander, H., Barton, V., Muangnoicharoen, S., Bray, P., Davies, J., Park, B., Wittlin, S., Brun, R., Preschel, M., Zhang, K. and Ward, S. (2010), Identification of a 1,2,4,5-Tetraoxane Antimalarial Drug-Development Candidate (RKA 182) with Superior Properties to the Semisynthetic Artemisinins. Angewandte Chemie International Edition, 49: 5693-5697. https://doi.org/10.1002/anie.201001026

2. Benoit-Vical, F.; Robert, A.; Meunier, B. In vitro and in vivo potentiation of artemisinin and synthetic endoperoxide antimalarial drugs by metalloporphyrins. Antimicrob. Agents Chemother. **2000**, 44, 2836–2841. DOI: 10.1128/AAC.44.10.2836-2841.2000

3. Bell, A. Antimalarial drug synergism and antagonism: Mechanistic and clinical significance. FEMS Microbiol. Lett. **2005**, 253, 171–184. doi: 10.1016/j.femsle.2005.09.035

4. L. Vivas, L. Rattray, L. B. Stewart, B. L. Robinson, B. Fugmann, R. K. Haynes, W. Peters, S. L. Croft Antimalarial efficacy and drug interactions of the novel semi-synthetic endoperoxide artemisone in vitro and in vivo Journal of Antimicrobial Chemotherapy, Volume 59, Issue 4, April 2007, Pages 658–665, https://doi.org/10.1093/jac/dkl563
5. D'Alessandro S, Camarda G, Corbett Y, Siciliano G, Parapini S, Cevenini L, Michelini E, Roda A, Leroy D, Taramelli D, Alano P. A chemical susceptibility profile of the *P. falciparum* transmission stages by complementary cell-based gametocyte assays. J Antimicrob Chemother. 2016; 71:1148-58. doi: 10.1093/jac/dkv493.
6. D'Alessandro S, Silvestrini F, Dechering K, Corbett Y, Parapini S, Timmerman M, Galastri L, Basilico N, Sauerwein R, Alano P, Taramelli D. A *Plasmodium falciparum* screening assay for anti-gametocyte drugs based on parasite lactate dehydrogenase detection. J Antimicrob Chemother. 2013 Sep;68(9):2048-58. doi: 10.1093/jac/dkt165.

7. Mortelmans, K.; Zeiger, E. The Ames Salmonella/microsome mutagenicity assay. *Mutat Res* **2000**, *455*, 29-60, doi:10.1016/s0027-5107(00)00064-6.

8. Mortelmans, K.; Riccio, E.S. The bacterial tryptophan reverse mutation assay with Escherichia coli WP2. *Mutat Res* **2000**, *455*, 61-69, doi:10.1016/s0027-5107(00)00076-2.

9. Chiu, P.J.; Marcoe, K.F.; Bounds, S.E.; Lin, C.H.; Feng, J.J.; Lin, A.; Cheng, F.C.; Crumb, W.J.; Mitchell, R. Validation of a [3H]astemizole binding assay in HEK293 cells expressing HERG K+ channels. *J Pharmacol Sci* **2004**, *95*, 311-319, doi:10.1254/jphs.fpe0040101.
